# Development of a Simple and Accurate Molecular Protocol Using 16SrRNA for Species-Specific Identification of *Achromobacter* spp.

**DOI:** 10.3390/pathogens14030271

**Published:** 2025-03-12

**Authors:** Giulia Maria Saitta, Laura Veschetti, Rebecca Feletti, Angela Sandri, Marzia Boaretti, Paola Melotti, Maria Carelli, Maria M. Lleò, Giovanni Malerba, Caterina Signoretto

**Affiliations:** 1 Diagnostic and Public Health Department, University of Verona, 37134 Verona, Italy; giuliamaria.saitta@univr.it (G.M.S.); rebecca.feletti01@universitadipavia.it (R.F.); angela.sandri@univr.it (A.S.); marzia.boaretti@univr.it (M.B.); 2 Infections and Cystic Fibrosis Unit, Division of Immunology, Transplantation and Infectious Diseases, IRCCS San Raffaele Scientific Institute, 20132 Milano, Italy; veschetti.laura@hsr.it; 3 Vita-Salute San Raffaele University, 20132 Milano, Italy; 4 General and Upper GI Surgery Division, Azienda Ospedaliera Universitaria Integrata Verona, Piazzale A. Stefani 1, 37126 Verona, Italy; 5 Cystic Fibrosis Centre, Azienda Ospedaliera Universitaria Integrata Verona, Piazzale A. Stefani 1, 37126 Verona, Italy; paola.melotti@aovr.veneto.it; 6 Cardiology Unit, IRCCS Azienda Ospedaliero-Universitaria di Bologna, 40138 Bologna, Italy; maria.carelli@aosp.bo.it; 7 GMLab, Department of Surgical Sciences, Dentistry, Gynaecology and Paediatrics, University of Verona, 37134 Verona, Italy; giovanni.malerba@univr.it

**Keywords:** *Achromobacter*, identification, cystic fibrosis, qPCR

## Abstract

The *Achromobacter* genus comprises 22 species and various genogroups. Some species with higher virulence or antibiotic resistance are more likely to cause chronic infections in people with cystic fibrosis (CF). Current identification methods often fail to accurately distinguish between the species or result in misidentifications due to biochemical similarities. This study aims to develop an accurate qPCR protocol for species-level identification that is applicable in clinical diagnostic laboratories. Whole-genome sequencing of clinical isolates from different *Achromobacter* species identified species-specific single-nucleotide polymorphisms (SNPs) in two 16S gene regions. Based on these SNPs, two sets of primers and qPCR probes were designed to generate unique identification profiles. Thermal profiles were optimized, and qPCR was performed on serial bacterial DNA dilutions to determine the detection limit (LOD). Four probes successfully identified three species: *A. xylosoxidans*, *A. dolens*, and *A. insuavis*. Two additional probes were designed for novel genotypes unrelated to publicly available sequences. The LOD ranged from 0.005 pg/µL to 1 pg/µL. Combined probes achieved 100% sensitivity, with specificity ranging from 97.95% to 100%. This qPCR protocol enables accurate species identification, overcoming the limitations of current methods, and represents a reliable tool for clinical diagnostics.

## 1. Introduction

*Achromobacter* spp. are Gram-negative bacilli broadly present in the environment, especially in moist soil, water sources, and plants [1]. *Achromobacter* spp. infections have been associated with various clinical conditions, including bacteriemia, meningitis, pneumonia, peritonitis and urinary tract infections [2,3]. In addition, *Achromobacter* spp. exhibit resistance to multiple classes of antibiotics [1,4,5].

Recently, *Achromobacter* spp. have gained attention as emerging opportunistic pathogens in cystic fibrosis (CF), a genetic disorder caused by mutations in the cystic fibrosis transmembrane conductance regulator (CFTR) gene [6]. These mutations result in a defective chloride transport in exocrine glands, leading to a thickened airway mucus, which fosters bacterial colonization [6]. While CF affects multiple organ systems, its primary cause of mortality is progressive lung disease, exacerbated by persistent infections with opportunistic pathogens such as *Staphylococcus aureus*, *Pseudomonas aeruginosa*, *Burkholderia cepacia complex*, *Stenotrophomonas maltophilia*, and *Achromobacter* spp. [7,8]. These infections contribute to lung inflammation, disease progression, and pulmonary impairment, ultimately increasing the risk of mortality [9,10].

Among these pathogens, *Achromobacter* spp. have been reported to cause chronic infections that exacerbate lung damage and respiratory decline [1,9,10,11,12], highlighting the critical need for improved ways of the identification and treatment of these opportunistic pathogens. The *Achromobacter* genus comprises 22 species and various genogroups. Among these, *Achromobacter xylosoxidans* is the most prevalent in CF, followed by *Achromobacter insuavis* and *Achromobacter dolens*. Other species associated with CF lung infections include *Achromobacter ruhlandii*, *Achromobacter dentifricans*, *Achromobacter insolitus*, and *Achromobacter aegrifacens* [13,14].

*Achromobacter* species exhibit regional variability in prevalence among CF patients. In Europe, *A. xylosoxidans* is the most frequently isolated species (36–65%, depending on the country), followed by *A. insuavis*, which has higher infection rates in Denmark (20–24%) and France (19%), and *A. dolens*, with lower rates compared to *A. insuavis* [2,15,16,17,18,19,20]. *A. xylosoxidans*, along with *A. ruhlandii*, *A. dolens*, and *A. insuavis*, is particularly adept at causing chronic infections in CF patients. These four species possess genes and mechanisms that facilitate long-term airway colonization, which can result in significant lung damage and functional decline. Indeed, up to half of CF patients with *A. xylosoxidans* colonization develop chronic infections, often accompanied by lung inflammation and respiratory deterioration [9,11,19,21,22,23,24], and a greater number of pulmonary exacerbation events and annual hospitalizations [1,12].

Recently, species-specific virulence and antibiotic resistance genetic profiles were reported, showing that some *Achromobacter* spp. are intrinsically resistant to several antibiotics, especially aminoglycosides, monobactams, tetracyclines, some penicillins, and cephalosporins [23,24,25]. Resistance to the most frequently used antimicrobial agents (e.g., trimethoprim–sulfamethoxazole, ceftazidime, piperacillin, and carbapenems) is on the rise, and there are no standard treatment protocols, requiring a case-by-case approach for treatment [23,26]. Considering this indication, accurate species identification could support clinical decisions. For example, *A. insuavis* is more sensitive to tigecycline (49%) compared to *A. xylosoxidans* (23%), while both species exhibit moderate sensitivity to ceftazidime–avibactam [27]. *A. rhulandii* shows low susceptibility to tigecycline, meropenem, and pieracillin–tazobactam, while *A. aegrifaciens* responds better to meropenem [27]. Without precise species identification, clinicians might select suboptimal antibiotics, potentially leading to treatment failure. Currently, methods for identifying *Achromobacter* species include biochemical testing (e.g., VITEK2), gene sequencing (such as *nrdA* gene encoding for ribonucleoside-diphosphate reductase 1 subunit alpha or 16S rRNA a ribosomal component found in all bacteria and archaea), multi-locus sequence typing (MLST), and matrix-assisted laser desorption/ionization time-of-flight mass spectrometry (MALDI-TOF MS). Biochemical testing remains the most commonly used method of routine bacterial identification in clinical laboratories due to its speed and reliability [28]. However, for *Achromobacter* spp., biochemical testing can sometimes result in inaccurate species identification due to similarities and, thus, difficult discrimination with other Gram-negative bacilli [23,28,29,30,31]. MALDI-TOF MS provides accurate genus-level identification but still faces challenges with *Achromobacter* species-level resolution due to the limited representation of species in databases [23]. Efforts to improve the accuracy of MALDI-TOF MS by expanding species databases have shown promise, but the results are still not satisfactory [31,32]. The limitations of these methods have already been highlighted both in clinical practice and in the literature: as shown by several studies, they can lead to false-positive and false-negative errors for *Achromobacter* spp. identification or to a limited species-level determination [33,34,35]. Sequence-based methods such as *nrdA* gene sequencing or MLST (which analyzes seven genes—*nusA*, *rpoB*, *eno*, *gltB*, *lepA*, *nuoL*, and *nrdA*—and is available via PubMLST) provide more accurate species identification [16,36]. However, nrdA gene sequencing requires bioinformatics tools for sequence analysis, while MLST, though simpler and faster than whole-genome sequencing (WGS), remains more costly and time-consuming than other molecular methods, such as qPCR. Although several methods and guidelines have been proposed to improve accurate identification, as previously described, differentiation of various species remains particularly challenging. This study aims to develop a reliable and accessible qPCR protocol for species-level identification of *Achromobacter* species using specific TaqMan probes (Eurofins Genomics, Ebersberg, Germany). This protocol could be widely applied in clinical diagnostic laboratories to support more informed decision-making in the management of CF patients.

## 2. Materials and Methods

### 2.1. Clinical Isolates

Fifty-two clinical isolates of *Achromobacter* spp. were collected from the sputum samples of patients at the CF Center of Verona, Italy. Informed consent was obtained according to projects CRCFC-CEPPO026 and CRCFC-CEPPO031, approved by the local Ethics Committee. The clinical isolates were recovered from twenty-six patients occasionally and chronically infected with *Achromobacter* spp. (according to the European Consensus Criteria or Leeds criteria); forty-one longitudinal isolates were collected from seventeen patients with chronic infections, while eleven strains were collected from nine patients with occasional infection. All strains isolated from each chronic patient were clonally related. Only one isolate was recovered from nine occasionally infected patients, while from two of these patients, we recovered two isolates; one of them harbored clonal isolates, while different clones were identified in the other one. A unique clonetype is considered to be the first isolate collected from a group of clonally related longitudinal isolates within the same patient, while subsequent isolates are considered to be clonally related variants of the original strain. The indications regarding the sampling timeframe are provided in Table S2. All the isolates were identified at the species level by whole-genome sequencing (WGS), followed by genome de novo assembly and phylogenetic analysis, as reported in our previous studies [14,37]. Strains were stored in Microbank (Pro-Lab Diagnostics, Neston, UK) at −80 °C.

### 2.2. Primers and Probes Design

Nucleotide sequences of 35 complete 16S ribosomal rRNA genes were retrieved from the de novo assemblies of the collection isolates, belonging to different species (*A. xylosoxidans*, *A. aegrifaciens*, *A. dolens*, *A. insolitus*, *and A. insuavis)* and two new genotypes (not phylogenetically related to any publicly available sequence) that we found in our collection. The 16S gene sequence from the *A. ruhlandii* reference genome (RefSeq accession: GCA_001051055.1) was also included in the dataset given the clinical relevance of this species [14,37]. Multiple-sequence alignment was performed using Clustalw 2.1 [38], and probes and primers were designed for the identification of *A. xylosoxidans*, *A. dolens*, *A. insuavis*, and the two new genogroups (*Achromobacter* NG). Probes and primers were designed using the software Primer3Plus 3.3.0 with default parameters (available at https://www.primer3plus.com/index.html, accessed on 8 October 2022). Candidate probes and primers were manually examined and selected to optimize the number of reactions needed to obtain the identification results. The specific probes were chosen with a Tm 5–10 °C higher than the Tm of the primers and a length of fewer than 30 nucleotides. Two sets of primers covering two distinct regions of the 16S gene harboring single-nucleotide polymorphisms (SNPs) and six probes were selected for experimental validation (Table 1). All the probes were designed as non-extendible hybridization probes, with FAM as reporter at the 5′ end and TAMRA as quencher at the 3′ end. The results obtained by the different qPCR must be associated, creating an identification profile specific for each *Achromobacter* species analyzed (Table 2).

### 2.3. DNA Extraction

*Achromobacter* spp. strains were isolated on Luria–Bertani (LB) agar (Merck, Darmstadt, Germany) and incubated at 37 °C for 48 h. We inoculated 1–2 of the obtained colonies in 5 mL of Brain Heart Infusion (BHI) culture medium (Merck, Darmstadt, Germany) and incubated them at 37 °C on a shaker for 16–18 h. A total of 0.5–1 mL of the bacterial suspension was centrifuged for 10 min at 5000× *g*. Genomic DNA was extracted within 1 h of collection using the QIAamp DNA Mini Kit (Qiagen, Milan, Italy) according to the manufacturer’s instructions. DNA was eluted in 100 µL of double-distilled water and stored at −20 °C. A NanoDrop 2000 UV/Vis Spectrophotometer (Thermo Fisher Scientific, Waltham, MA, USA) was used to evaluate DNA concentration and quality.

### 2.4. Real-Time PCR

The protocol used to analyze the different strains included a defined mixture of reagents (Table S1). Real-time PCR was carried out on a 7500 Fast Dx PCR system (Thermo Fisher Scientific, USA) with a first denaturation step at 95 °C for 2 min and 40 cycles of denaturation at 95 °C for 30 s; annealing and extension were performed in a single step at the same temperature at 65 °C for 30 s. All samples were tested in duplicate. The qPCR cut-off was redefined in each session, with manual setting of the threshold value as a function of the positive and negative control curves; in any case, the DeltaRn was between the values of 10^4^ and 10^5^.

## 3. Results

Fifty-two *Achromobacter* spp. clinical isolates were identified at the species level through WGS. The species distribution was as follows: 35 *A. xylosoxidans*, 4 *A. insuavis*, 4 *A. dolens*, 3 *A. aegrifacens*, 3 *A. insolitus*, and 3 *Achromobacter* NG [14,37]. Probes were tested on DNA extracted from these strains to assess analytical sensitivity and diagnostic sensitivity and specificity.

### 3.1. Analytical Sensitivity

For the analytical sensitivity test, the first qPCR was performed using a sequential 10-fold dilution of extracted DNA samples starting from 10^−2^ to 10^−7^ ng/µL for each probe. Furthermore, starting from the last 10-fold diluted concentration that can be detected using the qPCR protocol, we performed another qPCR experiment with 2-fold serial dilutions, from 2^−1^ to 2^−4^ g/µL. This was performed to determine the limit of detection (LOD) for each qPCR protocol, which ranged between 0.05 and 1 pg/µL. The lowest amount of DNA detectable for each probe is shown in Table 3.

### 3.2. Diagnostic Sensitivity and Specificity

Diagnostic sensitivity and specificity were assessed by testing each probe combination with all 52 different strains of our collection, and the identifications obtained by qPCR were compared with the actual WGS identification of the isolates (Table S2) [14,37].

The diagnostic sensitivity for each combination of the probes was assessed with the following Equation (1) [39]:

Diagnostic sensitivity = [True positive (TP)/(TP + False negative (FN))] × 100%,(1)

Sensitivity was 100% for all combinations of the probes (Table 4).

The diagnostic specificity for each probe was assessed with the following Equation (2) [39]:

Diagnostic specificity = [True negative (TN)/(TN + False positive (FP))] × 100%,(2)

Specificity ranged between 97.95% and 100%. The results obtained for each species are shown in Table 5.

The sensitivity and specificity calculated on the unique clonetypes (17 *A. xylosoxidans*, 2 *A. aegrifacens*, 1 *A. dolens*, 3 *A. insolitus*, 2 *A. insuavis*, and 2 *Achromobacter* NG) were 100% for all combinations of probes.

## 4. Discussion

In this study, a new qPCR-based method for species-specific identification of *Achromobacter* spp. was successfully developed, demonstrating 100% sensitivity and 97.95–100% specificity. By targeting SNP regions of the 16S rRNA gene, this approach addresses the persistent challenge of accurately identifying species within the genus—a critical issue given their distinct clinical relevance in people with CF. Compared with the existing literature, our molecular assay reveals advantages over previously described methods, highlighting its diagnostic potential.

Traditional biochemical testing methods, such as VITEK 2, are limited by the significant overlap of biochemical properties among *Achromobacter* species, often leading to misidentification [23,29]. While MALDI-TOF MS has been reported to improve genus-level identification [31,32], species-level resolution remains inconsistent due to limited representation in proteomic reference databases. Efforts to expand these databases have shown promise but remain constrained by the availability of comprehensive protein spectra for all species [20]. 16S rRNA sequencing is also time-consuming and laborious. In contrast, by focusing on species-specific SNPs, our qPCR assay overcomes these limitations and provides precise species differentiation, including identifying novel genotypes that were isolated in our CF center.

Our study leverages whole-genome sequencing (WGS) data for probe design, aligning with the approach taken in multi-locus sequence typing (MLST), as both methods utilize specific genetic markers to differentiate species based on their unique nucleotide sequences [40]. However, MLST is labor-intensive, expensive, and less feasible for routine clinical use, whereas our qPCR assay is simple, rapid, and cost-effective, making it more practical for diagnostic laboratories.

The qPCR method requires approximately one working day, including 3–4 h for bacterial DNA extraction and 40 min for the qPCR run, providing a direct and rapid result. In contrast, sequencing-based methods, while highly accurate, require time-consuming bioinformatic post-analysis. For instance, WGS involves multiple steps, such as sample preparation, library construction, and sequencing, with a total processing time of 3–5 days [41,42]. Similarly, MLST, although simpler and faster than WGS, remains costly and labor-intensive compared to other molecular techniques. On the other hand, the MALDI-TOF MS system enables extremely fast analysis (15–30 min) [43,44,45]. However, its ability to discriminate at the species level remains limited. Finally, the Vitek2 system, which involves sample preparation and analysis, takes significantly longer than other methods, requiring between 20 and 30 h [46,47,48]. Among these techniques, qPCR offers an optimal balance between speed, cost, and accuracy. It provides a direct result within hours, avoiding the lengthy processing times of sequencing methods while ensuring greater precision compared to MALDI-TOF. This makes qPCR a highly efficient choice for *Achromobacter* spp. identification in routine diagnostics (Table S3).

The diagnostic sensitivity of our assay (100%) is comparable to or exceeds that reported in studies using advanced sequence-based techniques. For example, Papalia et al. reported diagnostic inconsistencies using MALDI-TOF MS when identifying less common species like *A. dolens* [32], which our assay successfully differentiated. Furthermore, our ability to identify novel genotypes not phylogenetically related to any publicly available sequences suggests a broader applicability and adaptability to evolving genetic diversity within the genus.

Several studies have noted the difficulty of achieving both high specificity and inclusiveness in diagnostic assays for *Achromobacter* spp. [23,36]. Our qPCR assay demonstrated 97.95% specificity for *Achromobacter* NG; although this does not considerably impact the diagnostic accuracy, further probe refinement may enable the elimination of potential cross-reactivity with closely related species. These findings are in agreement with those of Fernández-Olmos et al. [29], who emphasized the importance of iterative optimization in diagnostic tool development.

Our findings align with recent studies highlighting the clinical importance of distinguishing *Achromobacter* species due to their varying virulence and antibiotic resistance profiles [13,25]. For instance, *A. xylosoxidans*, the most prevalent species in CF patients, has been strongly associated with chronic infections and significant respiratory decline [9,10]. Similarly, *A. dolens* and *A. insuavis* exhibit distinct geographical prevalence and infection patterns, as observed in Denmark and France [16,18,19,49]. By enabling accurate identification, our assay supports personalized treatment strategies tailored to the specific pathogen, aligning with the recommendations of Gabrielaite et al. for individualized CF management and Coward et al. for improved diagnostic precision in CF care [18].

We are aware, however, that this study has some limitations. First, the assay currently targets only a subset of the 22 known *Achromobacter* species, focusing on *A. xylosoxidans*, *A. dolens*, and *A. insuavis*, among the most frequent species, and novel genotypes of interest at our CF center. Expanding the probe set to include additional species will be essential for broader applicability. Additionally, the specificity for *Achromobacter* NG (97.95%) suggests potential cross-reactivity with closely related non-target species, warranting further probe refinement. Another potential limitation is the possible cross-reactivity with closely related species, which could reduce the assay’s specificity and potentially cause false-positive results. Despite our assay reaching very high sensitivity, we could only test a limited number of samples of *A. insuavis*, *A. dolens*, and *Achromobacter* NG due to the fact that *A. xylosoxidans* is the most prevalent species in CF.

In our study, we analyzed 52 isolates from 26 different patients. *Achromobacter* is often associated with chronic colonization in CF patients, so we considered it essential to validate the test under conditions where repeated sampling occurs; indeed, it is known that some genetic variation also occurs during chronic infection. In support of this, some previous studies on *Achromobacter* and other CF-related pathogens have included longitudinal isolates to assess the intra-patient variability and reliability of molecular identification methods over time [21,50]. To address potential concerns regarding data evaluation, we have also calculated sensitivity and specificity using only unique clonetypes, confirming that the results remain consistent. However, we believe that intra-patient variability justifies the inclusion of longitudinal isolates, as it allows for a more comprehensive assessment of the assay’s robustness in clinical setting.

Further testing on a higher number of samples belonging to these three species will be necessary for a full validation in clinical environment. Lastly, the assay’s reliance on high-quality DNA may pose challenges in a clinical environment where sample quality can vary, potentially impacting sensitivity. To address this, optimizing the clinical DNA extraction process is crucial to enhance sample quality and ensure the reliable application of this protocol.

The innovation of our qPCR-based method, compared to other existing qPCR approaches, such as the duplex-real time PCR assay by Price et al., lies in its ability to distinguish a subset of major *Achromobacter* species, reducing the risk of misclassification—an essential feature for targeted treatment decisions [51]. Our assay enables precise species differentiation, including novel genotypes, which is particularly relevant in CF care, where different *Achromobacter* species exhibit varying pathogenicity and antibiotic resistance profiles.

In contrast, the assay developed by Price et al. offers a more practical solution in some clinical settings, as it allows for the simultaneous detection of *Achromobacter* spp. and *A. xylosoxidans* with a streamlined workflow [51]. Their method benefits from a highly conserved target region (rpoB gene), reducing the likelihood of false negatives and making it applicable even in cases where DNA quality is suboptimal. However, while their approach is well-suited for rapid genus- and species-level screening, it does not achieve full species resolution, which may limit its usefulness in clinical decision-making where precise identification is necessary.

Despite requiring high-quality DNA and facing potential cross-reactivity with closely related species, our method remains a valuable tool for improved clinical management. The ability to accurately identify *Achromobacter* species at a refined level enhances diagnostic precision and supports more tailored therapeutic strategies, making it a significant advancement in the molecular identification of this pathogen. Based on this work, future studies could expand the assay to include additional *Achromobacter* species and validate its performance across larger, geographically diverse datasets. Integrating this method into multiplex diagnostic panels for CF pathogens may further enhance its utility. Additionally, longitudinal studies examining the impact of accurate species identification on treatment outcomes and resistance management are encouraged. In conclusion, our study aims to address a gap in the diagnostic landscape of *Achromobacter* spp., providing a practical, precise, and adaptable tool for clinical use.

## 5. Conclusions

Accurate species-level identification is crucial for understanding the specific pathogenic potential of different *Achromobacter* spp., particularly given the varying degrees of virulence and antimicrobial resistance among different species. The qPCR protocol developed in this study represents a step forward in addressing the challenges faced by clinicians in diagnosing *Achromobacter* spp. infections quickly and accurately. With its high sensitivity and specificity, this method could be implemented in clinical diagnostic laboratories to improve the speed and accuracy of diagnosis, ultimately leading to better-targeted therapies for critical patients such as those with CF. This is especially important as the incidence of *Achromobacter* infections in CF patients continues to rise, and timely, accurate identification is essential for optimal treatment.

## Figures and Tables

**Table 1:** Primers, probes, and species identification. NG: new genogroup.

Primers	Tm [°C]	GC Content [%]	Probes	Tm [°C]	GC Content [%]	Species
F1 TTGTAAAGCACTTTTGGCAG	53.2	40	Probe_A AGAAACGTCG(Y)GGGTTAATAC	58	45.2	*A. xylosoxidans* *A. dolens*
R1 CCAGTAATTCCGATTAACGC	55.3	45	Probe_B AGAAACGTCATGGGCTAATAC	58	43	*Achromobacter* NG
			Probe_C AGAAACGTCATGGGTTAATAC	58	38	*A. insuavis*
F2 CGGTGGATGATGTGGATTAA	55.3	45	Probe_1 AATGCCGAAGAGATTTGGCAGT	64	41	*A. xylosoxidans*
R2 GGACTTAACCCAACATCTCA	55.3	45	Probe_2 AATGCCGAAGAGATTTGGTAGT	60	41	*A. insuavis* *A. dolens*
			Probe_3 AATTCCGAAGAGATTTGGAAGT	60	36	*Achromobacter* NG

**Table 2:** *Achromobacter* species identification profiles.

Species		Identification	
*A. xylosoxidans*	Probe_A	Probe_1	
*A. insuavis*	Probe_C	Probe_2	
*A. dolens*	Probe_A	Probe_2	Probe_1 *
*Achromobacter NG*	Probe_B	Probe_3	

* Probe 1 is specific for *A. xylosoxidans*. A negative result is needed to confirm *A. dolens* identification.

**Table 3:** Limit of detention (LOD) of each probe.

Probes	LOD
Probe_A	0.275 pg/µL
Probe_B	0.1 pg/µL
Probe_C	1 pg/µL
Probe_1	0.05 pg/µL
Probe_2	0.05 pg/µL
Probe_3	0.05 pg/µL

**Table 4:** Results of diagnostic sensitivity for each combination of probes.

Species	Identification	Diagnostic Sensitivity	TP	FN
*A. xylosoxidans*	Probe_A + Probe_1	100%	35	0
*A. insuavis*	Probe_C + Probe_2	100%	4	0
*A. dolens*	Probe_A + Probe_2 + Probe_1	100%	4	0
*Achromobacter* NG	Probe_B + Probe_3	100%	3	0

**Table 5:** Results of diagnostic specificity for each combination of probes.

Species	Identification	Diagnostic Specificity	TN	FP
*A. xylosoxidans*	Probe_A + Probe_1	100%	17	0
*A. insuavis*	Probe_C + Probe_2	100%	48	0
*A. dolens*	Probe_A + Probe_2 + Probe_1	100%	48	0
*Achromobacter* NG	Probe_B + Probe_3	97.95%	48	1

## Data Availability

Data are available upon reasonable request to the corresponding authors.

## Supplementary Materials

The following supporting information can be downloaded at https://www.mdpi.com/article/10.3390/pathogens14030271/s1, Table S1: Reagents and concentration for the qPCR protocol; Table S2: WGS identification and qPCR identification; Table S3: Comparison of time efficiency between our method and other identification methods.

## Abbreviations

The following abbreviations are used in this manuscript:

CF	Cystic fibrosis
CFTR	Cystic fibrosis transmembrane conductance regulator
SNPs	Single-nucleotide polymorphisms
MLST	Multi-locus sequence typing
MALDI-TOF MS	Matrix-assisted laser desorption/ionization time-of-flight mass spectrometry
WGS	Whole-genome sequencing
LOD	Limit of detection
NG	New genogroup
TP	True positive
FN	False negative
TN	True negative
FP	False positive
