# Supplementary material for: Development of a Simple and Accurate Molecular Protocol Using 16SrRNA for Species-Specific Identification of Achromobacter spp
Source: Pathogens. 2025 Mar 12;14(3):271. doi: 10.3390/pathogens14030271 (PMC11945698; doi:10.3390/pathogens14030271)
Supplement: Supplementary file 1 [file pathogens-14-00271-s001.zip › pathogens-3470384-supplementary.pdf]

Supplementary Materials

Table S1. reagents and concentration for the qPCR protocol.

Reagents	Volume [µl]	Final Concentration
GoTaq® Probe qPCR Master mix	10	1X
Forward Primer	1	250 nM
Reverse Primer	1	250 nM
Probe	0.5	250 nM
DNA	5	≤ 250 ng
Nuclease-Free water	To a final volume of 20 µl	

Table S2. WGS identification and qPCR identification. The nomenclature used corresponds to the patient identifier (1 to 26), while the hyphenated numbers (-1, -2, etc.) denote isolates collected at different time points from the same patient. In column 2 (Time), the number of months elapsed from the previous sampling, with the first recorded sampling, is indicated. Na = not available.

ID	Time (months)	WGS identification	Probe_A	Probe_B	Probe_C	Probe_1	Probe_2	Probe_3	*qPCR identification*
1-1	/	*A. xylosoxidans*	+	−	−	+	+	−	*A. xylosoxidans*
1-2	4	*A. xylosoxidans*	+	−	−	+	+	−	*A. xylosoxidans*
2-1	/	*A. xylosoxidans*	+	−	−	+	+	+	*A. xylosoxidans*
2-2	3	*A. xylosoxidans*	+	−	−	+	+	−	*A. xylosoxidans*
2-3	2	*A. xylosoxidans*	+	−	−	+	+	−	*A. xylosoxidans*
2-4	2	*A. xylosoxidans*	+	−	−	+	+	−	*A. xylosoxidans*
3-1	/	*Achromobacter NG*	+	+	−	−	−	+	*Achromobacter NG*
3-3	10	*Achromobacter NG*	−	+	−	−	−	+	*Achromobacter NG*
4-1	/	*A. xylosoxidans*	+	−	−	+	+	−	*A. xylosoxidans*
4-2	3	*A. xylosoxidans*	+	−	−	+	+	−	*A. xylosoxidans*
4-3	6	*A. xylosoxidans*	+	−	−	+	+	−	*A. xylosoxidans*
5-1	/	*A. dolens*	+	+	−	−	+	−	*A. dolens*
5-2	5	*A. dolens*	+	+	−	−	+	−	*A. dolens*
5-3	2	*A. dolens*	+	+	−	−	+	−	*A. dolens*
5-4	2	*A. dolens*	+	+	−	−	+	−	*A. dolens*
6-1	/	*A. aegrifaciens*	−	−	+	−	−	+	Na
6-2	3	*A. aegrifaciens*	−	+	+	−	−	+	Na
7-1	/	*A. xylosoxidans*	+	−	−	+	+	−	*A. xylosoxidans*
7-2	1	*A. xylosoxidans*	+	−	−	+	+	−	*A. xylosoxidans*
8-1	/	*A. xylosoxidans*	+	−	−	+	+	−	*A. xylosoxidans*
8-2	39	*A. xylosoxidans*	+	−	−	+	+	−	*A. xylosoxidans*
9-1	/	*A. xylosoxidans*	+	−	−	+	+	−	*A. xylosoxidans*
9-2	4	*A. xylosoxidans*	+	−	−	+	+	−	*A. xylosoxidans*
9-3	2	*A. xylosoxidans*	+	−	−	+	+	−	*A. xylosoxidans*
9-4	1	*A. xylosoxidans*	+	−	−	+	+	−	*A. xylosoxidans*
9-5	0	*A. xylosoxidans*	+	−	−	+	+	−	*A. xylosoxidans*
9-6	3	*A. xylosoxidans*	+	−	−	+	+	−	*A. xylosoxidans*
10-1	/	*A. xylosoxidans*	+	−	−	+	+	−	*A. xylosoxidans*
10-2	4	*A. xylosoxidans*	+	−	−	+	+	−	*A. xylosoxidans*
10-3	12	*A. xylosoxidans*	+	−	−	+	+	−	*A. xylosoxidans*
10-4	13	*A. xylosoxidans*	+	−	−	+	+	−	*A. xylosoxidans*
10-5	10	*A. xylosoxidans*	+	−	−	+	+	−	*A. xylosoxidans*
10-6	3	*A. xylosoxidans*	+	−	−	+	+	−	*A. xylosoxidans*
11-1	/	*A. insuavis*	−	−	+	−	+	−	*A. insuavis*
11-2	2	*A. insuavis*	−	−	+	−	+	−	*A. insuavis*
11-3	8	*A. insuavis*	+	−	+	−	+	−	*A. insuavis*
12-1	/	*A. xylosoxidans*	+	−	−	+	+	−	*A. xylosoxidans*
12-2	5	*A. aegrifaciens*	−	−	+	−	−	−	Na
13-1	/	*A. xylosoxidans*	+	−	−	+	+	−	*A. xylosoxidans*
14-1	/	*A. insolitus*	+	−	−	−	−	+	Na
15-1	/	*A. insuavis*	−	−	+	−	+	−	*A. insuavis*
16-1	/	*Achromobacter NG*	−	−	+	−	−	+	*Achromobacter NG*
17-1	/	*A. xylosoxidans*	+	−	−	+	+	−	*A. xylosoxidans*
18-1	/	*A. insolitus*	+	−	−	−	−	−	Na

19-1	/	*A. xylosoxidans*	+	−	−	+	+	−	*A. xylosoxidans*
20-1	/	*A. insolitus*	+	−	−	−	−	−	Na
21-1	/	*A. xylosoxidans*	+	−	−	+	+	−	*A. xylosoxidans*
22-1	/	*A. xylosoxidans*	+	−	−	+	+	−	*A. xylosoxidans*
23-1	/	*A. xylosoxidans*	+	−	−	+	+	+	*A. xylosoxidans*
24-1	/	*A. xylosoxidans*	+	−	−	+	+	−	*A. xylosoxidans*
25-1	/	*A. xylosoxidans*	+	−	−	+	+	−	*A. xylosoxidans*
26-1	/	*A. xylosoxidans*	+	−	−	+	+	−	*A. xylosoxidans*

Table S3. Comparison of time efficiency between our method and other identification methods.

Identification Method	Operations	Time	Citations
qPCR	DNA extraction, qPCR	3–4 hours	
WGS	Sample preparation, library construction, sequencing	3–5 days	[42,43]
MALDI–TOF MS	Sample preparation, analysis	15–30 minutes	[44–46]
Vitek2	Sample preparation, analysis	20–30 hours	[47–49]
